# Development of Melanoma and Other Nonkeratinocyte Skin Cancers After Thyroid Cancer Radiation

**DOI:** 10.1001/jamanetworkopen.2024.34841

**Published:** 2024-09-19

**Authors:** Shawheen J. Rezaei, Michael L. Chen, Jiyeong Kim, Esther M. John, John B. Sunwoo, Eleni Linos

**Affiliations:** 1 Department of Dermatology, Stanford University School of Medicine, Stanford, California; 2 Center for Digital Health, Stanford University, Stanford, California; 3 Department of Epidemiology and Population Health, Stanford University School of Medicine, Stanford, California; 4 Department of Medicine (Oncology), Stanford University School of Medicine, Stanford, California; 5 Stanford Cancer Institute, Stanford University School of Medicine, Stanford, California; 6 Department of Otolaryngology—Head and Neck Surgery, Stanford University School of Medicine, Stanford, California

## Abstract

This cohort study evaluates the association between thyroid malignant neoplasms and melanoma and other nonkeratinocyte skin cancers.

## Introduction

Thyroid cancer and skin cancer are commonly diagnosed cancers in the US.^1^ Prior research has suggested a link between papillary thyroid cancer and malignant melanoma via common pathogenic variants.^2^ Unlike nonmelanoma skin cancers, the evidence for a relationship between melanoma and exposure to ionizing radiation is sparse.^3^ Although radioactive iodine therapy for thyroid cancer is thought to be associated with subsequent solid organ and hematologic tumors, the association has rarely been studied.^4,5^ Thus, we characterized the risk of subsequent melanoma and other nonkeratinocyte skin cancers in patients with primary thyroid malignant neoplasms.

## Methods

Our analysis employed US population-based data from 17 cancer registries made available through the Surveillance, Epidemiology, and End Results (SEER) Program of the National Cancer Institute. We included patients with thyroid cancer diagnosed from 2000 to 2019 and followed up through 2020 for subsequent cancers. Standardized incidence ratios (SIRs) were calculated as the incidence of an event observed to occur in a specific population divided by the incidence of an event expected to occur in a standard population. We computed SIRs to estimate the risk for subsequent cancer development, and adjusted them by sex, self-reported race and ethnicity (Hispanic [all races], Non-Hispanic American Indian/Alaska Native, Non-Hispanic Asian or Pacific Islander, Non-Hispanic Black, Non-Hispanic White, and Non-Hispanic unknown race), age, and year of initial diagnosis (as determined by the SEER database; these variables, including self-reported race and ethnicity, were used in the development of SIRs by the SEER*Stat software). This study leveraged SIRs to quantify the risk of nonkeratinocyte skin cancer (ie, melanoma or other) diagnoses after primary thyroid cancer diagnosis in relation to type of radiation treatment recorded in SEER. Keratinocyte skin cancers (ie, basal and squamous cell carcinomas) were excluded since these cancers are not documented in the SEER registries. The Stanford institutional review board deemed this study exempt from review and the need for informed consent due to the use of deidentified data. The study followed the Strengthening the Reporting of Observational Studies in Epidemiology (STROBE) reporting guidelines. Statistical analyses were completed using SEER*Stat software version 8.4.3 (Surveillance Research Program) from January 2024 to April 2024 with a *P *value less than .05 considered significant. Further detail of the methods and nonkeratinocyte skin cancer subtypes included in the analysis are provided in the eMethods in Supplement 1.

## Results

Of the 174 916 patients (75 134 female [78.8%]; 15 653 [16.4%] Hispanic [all races], 9836 [10.3%] non-Hispanic Asian or Pacific Islander, and 612 [64.6%] non-Hispanic White) with primary thyroid cancer diagnosed from 2000 to 2019, 79 576 (45.5%) had first-course treatment of some form of radiation (Table 1). A total of 865 nonkeratinocyte skin cancers (790 melanoma) were diagnosed following thyroid cancer, and, of those, 171 (19.8%) were located on the skin of the head or neck. Notably, when limiting the cancer site to the head and neck skin, the SIRs were higher than expected following thyroid cancer treated with radioactive iodine for all nonkeratinocyte skin cancers (SIR, 1.64; 95% CI, 1.32-2.02), melanoma (SIR, 1.56; 95% CI, 1.22-1.97) and other nonkeratinocyte skin cancers (SIR, 2.07; 95% CI, 1.23-3.27) (Table 2). The risk of head and neck skin cancer was not elevated in patients who did not receive radioactive iodine therapy. The SIR was statistically significant if the primary thyroid cancer treated with any type of radiation was the papillary subtype (SIR, 1.69; 95% CI, 1.35-2.09) but not for other thyroid cancer subtypes, likely due to insufficient sample sizes of other subtypes.

**Table 1.** Characteristics of Patients Diagnosed With Thyroid Cancer From 2000 to 2019, by Radiation Therapy

Characteristics (as identified by SEER database)	Patients, No. (%)		
	No radiation therapy/unknown (n = 95 340)	Radiation therapy (n = 79 576)	Total (N = 174 916)
Sex			
Male	20 206 (21.2)	20 544 (25.8)	40 750 (23.3)
Female	75 134 (78.8)	59 032 (74.2)	134 166 (76.7)
Race and ethnicity			
Hispanic (all races)	15 653 (16.4)	14 702 (18.5)	30 355 (17.4)
Non-Hispanic American Indian/Alaska Native	472 (0.5)	445 (0.6)	917 (0.5)
Non-Hispanic Asian or Pacific Islander	9836 (10.3)	9480 (11.9)	19 316 (11)
Non-Hispanic Black	6624 (7)	4314 (5.4)	10 938 (6.3)
Non-Hispanic White	61 612 (64.6)	50 218 (63.1)	111 830 (63.9)
Non-Hispanic unknown race	1143 (1.2)	417 (0.5)	1560 (0.9)
Age at diagnosis, y			
0-19	1672 (1.8)	2127 (2.7)	3799 (2.2)
20-39	25 054 (26.3)	24 347 (30.6)	49 401 (28.2)
40-59	43 422 (45.5)	35 668 (44.8)	79 090 (45.2)
60-79	22 543 (23.6)	16 096 (20.2)	38 639 (22.1)
≥80	2649 (2.8)	1338 (1.7)	3987 (2.3)
Neighborhood SES, quintile^a^			
1 (lowest)	59 846 (33.4)	7264 (10.5)	106 270 (33.5)
2	17 290 (9.6)	9364 (13.6)	30 596 (9.6)
3	5206 (2.9)	11 732 (17)	9014 (2.8)
4	3879 (2.2)	15 772 (22.8)	6919 (2.2)
5 (highest)	3372 (1.9)	21 319 (30.9)	5820 (1.8)
Unknown	89 593 (50)	3575 (5.2)	158 619 (50)
Rural vs urban^b^			
Urban	46 424 (67.3)	59 846 (66.8)	106 270 (67)
Mostly urban	13 306 (19.3)	17 290 (19.3)	30 596 (19.3)
Mostly rural	3808 (5.5)	5206 (5.8)	9014 (5.7)
Rural	3040 (4.4)	3879 (4.3)	6919 (4.4)
Unknown	2448 (3.5)	3372 (3.8)	5820 (3.7)
Time period of diagnosis (by decade)			
2000-2009	33 584 (35.2)	35 403 (44.5)	68 987 (39.4)
2010-2019	61 756 (64.8)	44 173 (55.5)	105 929 (60.6)

Abbreviations: SEER, Surveillance, Epidemiology, and End Results; SES, socioeconomic status.

^a^ Composite SES scores were developed from 7 SES variables and stratified to quintiles based on 2010 census tracts; data limited to 2006 to 2018.

^b^ Census-based Urban Rural Indicator Code using 2010 census tracts; data limited to 2006 to 2018.

**Table 2.** Standardized Incidence Ratios (SIRs) for Subsequent Nonkeratinocyte Skin Cancer After Thyroid Cancer From 2000 to 2020, by Radiation Treatment Type and Site of Subsequent Cancer

Skin cancer site, type, and radiation recode	Patients, No.		SIR (95% CI)	Excess risk, No. per 100 000 persons per year
	Observed	Expected		
**All skin**				
None/unknown				
Total	434	332.44	1.31 (1.19-1.43)^a^	13.6
Melanoma	399	309.16	1.29 (1.17-1.42)^a^	12.1
Other	35	23.28	1.50 (1.05-2.09)^a^	1.6
Beam radiation				
Total	9	9.52	0.94 (0.43-1.79)	−2.9
Melanoma	8	8.76	0.91 (0.39-1.8)	−4.2
Other	1	0.77	1.3 (0.03-7.24)	1.3
Radioactive implants				
Total	5	5.89	0.85 (0.28-1.98)	−6.5
Melanoma	5	5.47	0.91 (0.3-2.13)	−3.5
Other	0	0.41	0 (0-8.97)	−3
Radioisotopes				
Total	402	282.5	1.42 (1.29-1.57)^a^	17.4
Melanoma	364	263.73	1.38 (1.24-1.53)^a^	14.6
Other	38	18.77	2.02 (1.43-2.78)^a^	2.8
Combination of beam with implants or isotopes				
Total	1	2.65	0.38 (0.01-2.1)	−33.7
Melanoma	0	2.44	0 (0-1.51)	−49.9
Other	1	0.21	4.84 (0.12-26.95)	16.2
Radiation, NOS method or source not specified				
Total	2	1.76	1.13 (0.14-4.1)	5.4
Melanoma	2	1.64	1.22 (0.15-4.41)	8.3
Other	0	0.13	0 (0-29.25)	−2.9
Refused				
Total	3	1.83	1.64 (0.34-4.79)	26
Melanoma	3	1.69	1.77 (0.37-5.18)	29
Other	0	0.14	0 (0-27.18)	−3
Recommended, unknown if administered				
Total	9	6.08	1.48 (0.68-2.81)	19.5
Melanoma	9	5.66	1.59 (0.73-3.02)	22.4
Other	0	0.42	0 (0-8.75)	−2.8
**Head/neck**				
None/unknown				
Total	73	65.25	1.12 (0.88-1.41)	1
Melanoma	60	54.17	1.11 (0.85-1.43)	0.8
Other	13	11.08	1.17 (0.62-2.01)	0.3
Beam radiation				
Total	2	2.27	0.88 (0.11-3.18)	−1.5
Melanoma	2	1.88	1.07 (0.13-3.85)	0.7
Other	0	0.4	0 (0-9.29)	−2.2
Radioactive implants				
Total	1	1.18	0.85 (0.02-4.71)	−1.3
Melanoma	1	0.99	1.01 (0.03-5.64)	0.1
Other	0	0.19	0 (0-18.95)	−1.4
Radioisotopes				
Total	89	54.25	1.64 (1.32-2.02)^a^	5.1
Melanoma	71	45.56	1.56 (1.22-1.97)^a^	3.7
Other	18	8.69	2.07 (1.23-3.27)^a^	1.4
Combination of beam with implants or isotopes				
Total	1	0.64	1.56 (0.04-8.69)	7.3
Melanoma	0	0.53	0 (0-6.9)	−10.9
Other	1	0.11	9.37 (0.24-52.2)	18.3
Radiation, NOS method or source not specified				
Total	2	0.36	5.6 (0.68-20.23)	37.5
Melanoma	2	0.3	6.74 (0.82-24.35)	38.9
Other	0	0.06	0 (0-61.14)	−1.4
Refused				
Total	2	0.37	5.42 (0.66-19.6)	36.2
Melanoma	2	0.3	6.59 (0.8-23.79)	37.6
Other	0	0.07	0 (0-56.73)	−1.4
Recommended, unknown if administered				
Total	1	1.21	0.83 (0.02-4.6)	−1.4
Melanoma	1	1.01	0.99 (0.03-5.5)	−0.1
Other	0	0.2	0 (0-18.56)	−1.3

^a^ Significant at *P* < .05.

## Discussion

We found elevated risk of melanoma and other nonkeratinocyte skin cancers in patients with primary thyroid cancer who received radioactive iodine therapy, particularly in the head and neck region. Radioactive iodine therapy is a mainstay treatment for thyroid cancer with associated reductions in all-cause and cancer-specific mortality.^6^ Although the risks of subsequent cancer do not outweigh the benefits of treatment, our findings suggest that patients treated for thyroid cancer may benefit from follow-up skin cancer screening.

Limitations of the study include small sample sizes of nonpapillary thyroid cancers and non-White race and ethnicity, potential confounding characteristics not measured, possible sampling bias, and limited treatment data available in SEER. Further research is needed to identify the mechanisms through which thyroid cancer and thyroid cancer therapy are associated with skin cancer, particularly if certain thyroid cancer subtypes are associated with elevated subsequent malignancy risk.

## Notes

Supplement 1.
eMethods.
**eTable.** Other Nonkeratinocyte Skin Cancer Subtypes After Primary Thyroid Cancer, Diagnosed From 2000-2020

Supplement 2.
Data Sharing Statement
