# Supplementary material for: Development of Melanoma and Other Nonkeratinocyte Skin Cancers After Thyroid Cancer Radiation
Source: JAMA Netw Open. 2024 Sep 19;7(9):e2434841. doi: 10.1001/jamanetworkopen.2024.34841 (PMC11413709; doi:10.1001/jamanetworkopen.2024.34841)
Supplement: Supplement 1. — eMethods. eTable. Other Nonkeratinocyte Skin Cancer Subtypes After Primary Thyroid Cancer, Diagnosed From 2000-2020 [file jamanetwopen-e2434841-s001.pdf]

Supplemental Online Content

Rezaei SJ, Chen ML, Kim J, John EM, Sunwoo JB, Linos E. The development of melanoma and other nonkeratinocyte skin cancers after thyroid cancer radiation. *JAMA Netw Open*. 2024;7(9):e2434841. doi:10.1001/jamanetworkopen.2024.34841

eMethods.
eTable. Other Nonkeratinocyte Skin Cancer Subtypes After Primary Thyroid Cancer, Diagnosed From 2000 to 2020

This supplemental material has been provided by the authors to give readers additional information about their work.

© 2024 Rezaei et al. *JAMA Network Open.*

eMethods

The Multiple Primary-Standardized Incidence Ratios (MP-SIR) session was used to conduct the statistical analysis using SEER*Stat software (version 8.4.3, Surveillance Research Program, National Cancer Institute, Bethesda, MD, USA). Statistical significance was determined by a p-value <0.05. Radiation treatment that was listed as radioisotope in SEER was considered radioactive iodine therapy. Cancer diagnoses were identified using the International Classification of Diseases for Oncology, third edition (ICD-O-3) Site Recode/ World Health Organization (WHO) 2008 Definition. The analysis was performed January 2024 to April 2024. The Stanford IRB deemed this study exempt from review due to the use of only deidentified data. Subtypes included in our study are as follows:

eTable. Other Nonkeratinocyte Skin Cancer Subtypes After Primary Thyroid Cancer, Diagnosed From 2000 to 2020

ICD-O-3 code
8247/3: Merkel cell carcinoma
8410/3: Sebaceous adenocarcinoma
8832/3: Dermatofibrosarcoma, NOS
8413/3: Eccrine adenocarcinoma
8200/3: Adenoid cystic carcinoma
8402/3: Nodular hidradenoma, malignant
8800/3: Sarcoma, NOS
8390/3: Skin appendage carcinoma
8480/3: Mucinous adenocarcinoma
8542/3: Paget disease, extramammary (except Paget disease of bone)
8890/3: Leiomyosarcoma, NOS
8255/3: Adenocarcinoma with mixed subtypes
8407/3: Sclerosing sweat duct carcinoma
8430/3: Mucoepidermoid carcinoma
8830/3: Malignant fibrous histiocytoma
8833/3: Pigmented dermatofibrosarcoma protuberans
9120/3: Hemangiosarcoma

© 2024 Rezaei et al. *JAMA Network Open.*
