# Supplementary material for: Development of Melanoma and Other Nonkeratinocyte Skin Cancers After Thyroid Cancer Radiation
Source: JAMA Netw Open. 2024 Sep 19;7(9):e2434841. doi: 10.1001/jamanetworkopen.2024.34841 (PMC11413709; doi:10.1001/jamanetworkopen.2024.34841)
Supplement: Supplement 2. — Data Sharing Statement [file jamanetwopen-e2434841-s002.pdf]

Data Sharing Statement

Rezaei. The Development of Melanoma and Other Nonkeratinocyte Skin Cancers After Thyroid Cancer Radiation. *JAMA Netw Open*. Published September 19, 2024.
doi:10.1001/jamanetworkopen.2024.34841

Data
Data available: No

Additional Information
Explanation for why data not available: This article uses a publicly available data source, the CDC's SEER registry, for the analysis
